# Discordant localization of WFA reactivity and brevican/ADAMTS-derived fragment in rodent brain

**DOI:** 10.1186/1471-2202-9-14

**Published:** 2008-01-25

**Authors:** Joanne M Ajmo, Autumn K Eakin, Michelle G Hamel, Paul E Gottschall

**Affiliations:** 1 Department of Molecular Pharmacology and Physiology, University of South Florida College of Medicine, Tampa, Florida USA; 2 Department of Pharmacology and Toxicology, University of Arkansas for Medical Sciences, Little Rock, Arkansas USA

## Abstract

**Background:**

Proteoglycan (PG) in the extracellular matrix (ECM) of the central nervous system (CNS) may act as a barrier for neurite elongation in a growth tract, and regulate other characteristics collectively defined as structural neural plasticity. Proteolytic cleavage of PGs appears to alter the environment to one favoring plasticity and growth. Brevican belongs to the lectican family of aggregating, chondroitin sulfate (CS)-bearing PGs, and it modulates neurite outgrowth and synaptogenesis. Several ADAMTSs (a disintegrin and metalloproteinase with thrombospondin motifs) are glutamyl-endopeptidases that proteolytically cleave brevican. The purpose of this study was to localize regions of adult CNS that contain a proteolytic-derived fragment of brevican which bears the ADAMTS-cleaved neoepitope sequence. These regions were compared to areas of *Wisteria floribunda *agglutin (WFA) reactivity, a common reagent used to detect "perineuronal nets" (PNNs) of intact matrix and a marker which is thought to label regions of relative neural stability.

**Results:**

WFA reactivity was found primarily as PNNs, whereas brevican and the ADAMTS-cleaved fragment of brevican were more broadly distributed in neuropil, and in particular regions localized to PNNs. One example is hippocampus where the ADAMTS-cleaved brevican fragment is found surrounding pyramidal neurons, in neuropil of stratum oriens/radiatum and the lacunosum moleculare. The fragment was less abundant in the molecular layer of the dentate gyrus. Mostly PNNs of scattered interneurons along the pyramidal layer were identified by WFA. In lateral thalamus, the reticular thalamic nucleus stained abundantly with WFA whereas ventral posterior nuclei were markedly immunopositive for ADAMTS-cleaved brevican. Using Western blotting techniques, no common species were reactive for brevican and WFA.

**Conclusion:**

In general, a marked discordance was observed in the regional localization between WFA and brevican or the ADAMTS-derived N-terminal fragment of brevican. Functionally, this difference may correspond to regions with varied prevalence for neural stability/plasticity.

## Background

**Figure 1:**
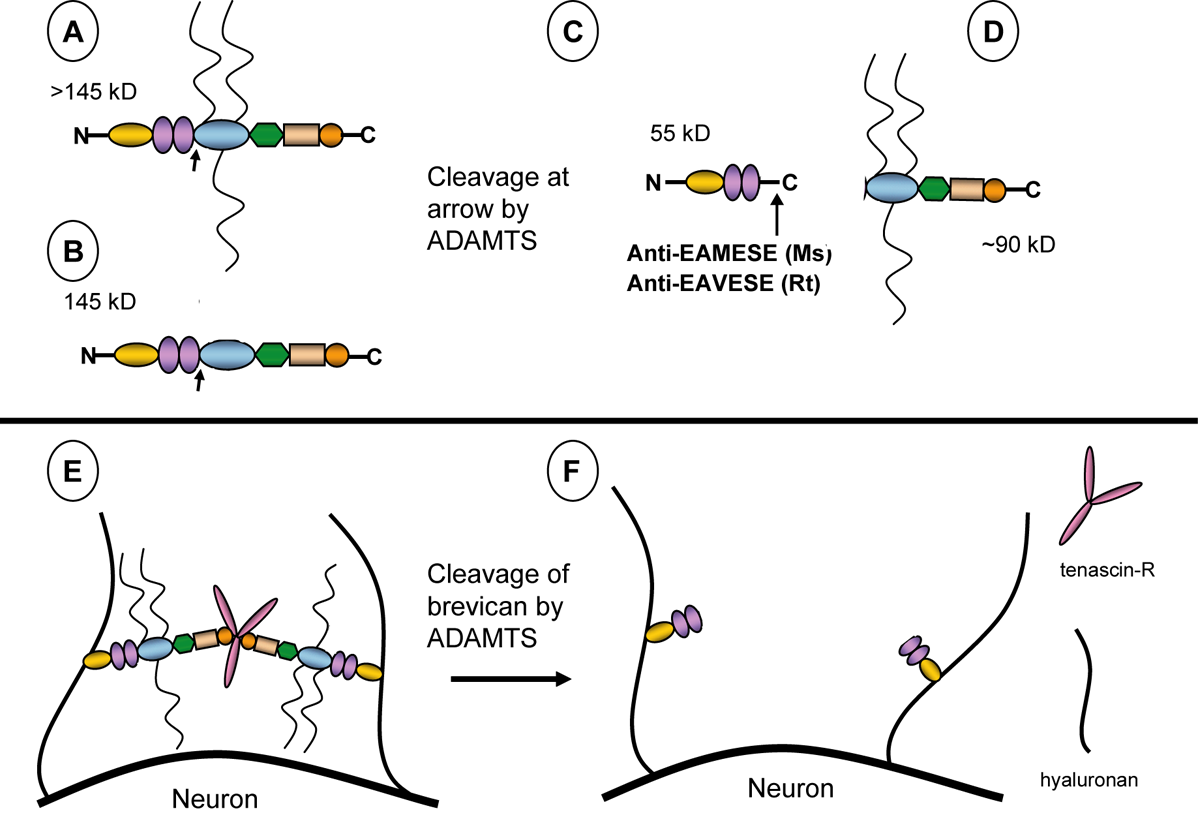
**Brevican processing and fragments formed by ADAMTS cleavage**. Schematic representation of brevican, its endogenous proteolytic fragments and their interaction with other components of brain ECM: (A) Secreted brevican core protein which bears 1–3 chondroitin sulfate chains (MW > 145 kD). (B) Secreted brevican core protein without chondroitin sulfate chains (MW = 145 kD). When cleaved by extracellular glutamyl-endopeptidases, the ADAMTSs (arrows in A and B), an N-terminal, 55 kD fragment is formed (C) that contains a unique C-terminal murine (Ms) epitope sequence "EAMESE", homologous to the rat (Rt) "EAVESE" which are selectively recognized by respective neoepitope antibodies, anti-EAMESE or anti-EAVESE (C). A larger C-terminal fragment is formed upon ADAMTS cleavage (D). The > 145 kD and 145 kD isoforms of brevican or other lecticans in matrix form an tertiary aggregate complex with hyaluronan and tenascin-R (E) and when cleaved by ADAMTSs, the proteolytic degradation of brevican "loosens" the ECM complex (F).

Extracellular matrix (ECM) in the central nervous system (CNS) is deposited in the extracellular space of the neuropil and around a subset of neurons in the form of distinctive perineuronal nets (PNNs), coverings of matrix that ensheath perikarya, proximal dendrites and axon initial segments. The components of this matrix are aggregating proteoglycans (PGs), termed lecticans, that interact with tenascin and hyaluronan to form complexes which maintain an anionic environment in the extracellular milieu of the CNS [1,2] (Fig. 1D). The core proteins of lecticans bear covalently-linked, highly negatively-charged, linear chondroitin sulfate (CS) chains that consist of glucuronic acid/N-acetylgalactosamine repeats, sulfated to varying extents at the 4 and 6 positions [3]. Methods developed to detect the lecticans in fixed brain sections have employed: 1) antibodies that recognize CS epitopes of the lecticans, 2) antibodies that recognize initial disaccharides of CS chains exposed on the core protein after digestion with chondroitinase ABC (Chase), 3) labeled lectins that recognize "selective" monosaccharide components of CS, and 4) antibodies that recognize the core protein.

*Wisteria floribunda *agglutinin (WFA) is a lectin that binds to terminal N-acetylgalactosamine residues [4] and decorates various structures, including PNNs in the CNS, where its reactivity has been well-documented [5-7]. Various rostral-caudal layers of rat cerebral cortex, and particularly the retrosplenial cortex, thalamus, cerebellum and brain stem are regions that contain numerous PNNs prominently labeled by WFA [6]. In rat neocortex, several types of functional morphology are associated with WFA-reactive PNNs [5,8]. Some data indicate that lectin binding identifies terminal N-acetylgalactosamine present on neuronal cell surface glycoproteins [9], whereas others suggest the reactivity seen with WFA may detect CS directly. Importantly, WFA binding in nervous tissue co-localizes with signal from antibodies raised against CSs [10], and signal is lost when tissue sections are pre-treated with Chase [11], suggesting that WFA binds indirectly or directly to CS. Thus, WFA reactivity has become a standard method of identifying CS-containing subsets of neurons in the CNS that are surrounded by PNNs.

The lecticans, including brevican, aggrecan and the V2 isoform of versican, are highly expressed in the adult brain. The deposition of these lecticans is heterogeneous in the complex ECM of PNNs and in the neuropil [3] (Fig. 1E). Functionally, CS side chains of the lecticans inhibit neurite elongation and even may stabilize synapses in neural networks [3,12,13]. Among the lecticans, brevican is highly abundant in brain, where various isoforms are found including a > 145 kD molecule that carries 1–3 CS chains (Fig. 1A), a core 145 kD protein without CS (Fig. 1B), a 120 kD glycosylphosphatidylinositol-linked membrane-bound form, and 55 kD N-terminal, and 80 kD C-terminal fragments (Fig. 1C, 1D) that are the result of endopeptidase cleavage of the holoprotein. The proteases mainly responsible for cleavage of brevican are glutamyl-endopeptidases, the ADAMTSs (adisintegrin and metalloproteinase with thrombospondin motifs) (Fig. 1E, 1F). Several of these multi-domain proteases [14] are expressed in brain (ADAMTS1, 4, 5, and 9) [15-17] (our unpublished observations) and cleave aggrecan [18], versican [19] and brevican [20] (for review, see [21]). ADAMTS-cleaved fragments of each lectican are found in untreated nervous tissue extracts [16,19,20,22-24], indicating that ADAMTSs are active proteases capable of cleaving lecticans in a "normal" nervous system. Fragments of lecticans may be localized in brain tissue sections by using antibodies raised against the terminal, neoepitope sequence of the core protein that is exposed after ADAMTS cleavage [20,21,25,26]. Using an antibody that recognizes the C-terminal residues (EAVESE) that are uncovered after ADAMTS-induced release of the N-terminal fragment of brevican [20], we noted that the distribution of this immunoreactivity in rat hippocampus was markedly different from WFA reactivity in the same region. We expected that the distribution of the signal from both reagents would be similar, since both N- and C-terminal fragments of brevican appear to be stable after cleavage [3], and the preponderance of the C-terminal fragments bear CS chains. Thus, the purpose of this study was to describe the distribution and characteristic immunoreactivity for the ADAMTS-cleaved fragment of brevican, and compare this with WFA binding in the rodent CNS. The results show a marked discordance between the two, with the breadth of distribution of the ADAMTS-derived brevican fragment being much wider than that of WFA reactivity.

## Results

**Figure 2:**
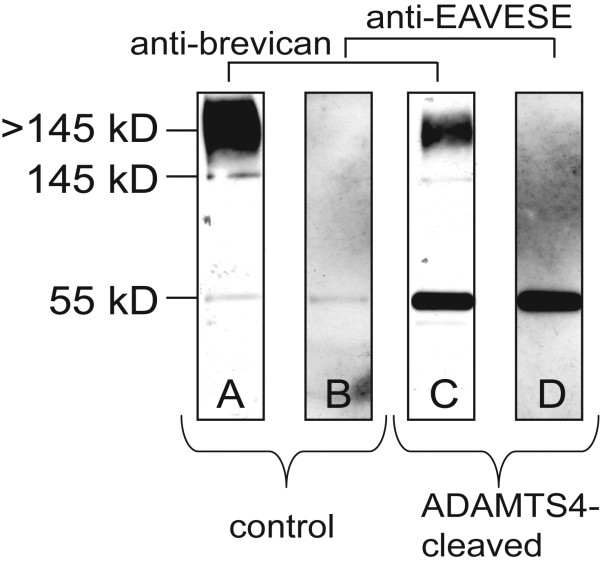
**N-terminal and neoepitope fragment formed by ADAMTS cleavage of brevican**. Recombinant ADAMTS4 cleavage product of rat brevican is recognized by anti-EAVESE: (A) Proteoglycan-containing fraction eluates of rat brain extracts from a DEAE cation exchange matrix were probed with anti-brevican. (B) Proteoglycan fraction was probed with anti-EAVESE, antibody raised against the C-terminal neoepitope sequence of the ADAMTS-cleaved N-terminal fragment of brevican. (C) Proteoglycan fraction after incubation with recombinant ADAMTS4 and probed with anti-brevican or (D) anti-EAVESE. After incubation with human recombinant ADAMTS4, brevican was proteolytically cleaved resulting in diminished full length brevican and the appearance of a 55 kD N-terminal fragment recognized by both anti-brevican and anti-EAVESE.

Brevican exists in rodent brain ECM as a holoprotein, in part, with a central region that bears CS chains and globular terminal domains which do not. When brevican is detected on Western blot using an N-terminal region antibody, a 55 kD, N-terminal fragment of brevican is prominent. The predominant protease responsible for this cleavage is ADAMTS-derived, glutamyl-endopeptidase activity. The neoepitope antibody(s) used in these experiments, anti-EAV(M)ESE, represents the C-terminal sequence exposed on the N-terminal, 55 kD fragment of brevican after ADAMTS cleavage [20] (Fig. 1). To verify that this antibody recognizes ADAMTS-cleaved brevican, the CS-bearing form of brevican was partially purified on a DEAE anion exchange matrix, and the PG-containing eluant was incubated with active human recombinant ADAMTS4. As shown in Fig. 2, when a Western blot of the DEAE extract was probed using an N-terminal region monoclonal, anti-brevican antibody, a smear > 145 kD was observed (Fig. 2A). After digestion by ADAMTS4, the abundance of the holoprotein was markedly reduced with the appearance of a 55 kD N-terminal fragment (Fig. 2C). When probed with polyclonal anti-EAVESE, little or no anti-EAVESE immunoreactive fragment was present in the DEAE extract prior to digestion (since the fragment does not bind the column) (Fig. 2B), however, after cleavage of brevican by human recombinant ADAMTS4, the same 55 kD fragment was apparent (Fig. 2D). This indicates that N-terminal, ADAMTS-cleaved brevican is recognized by anti-EAVESE as a 55 kD, N-terminal fragment, the same fragment detected using a general N-terminal region anti-brevican antibody.

**Figure 3:**
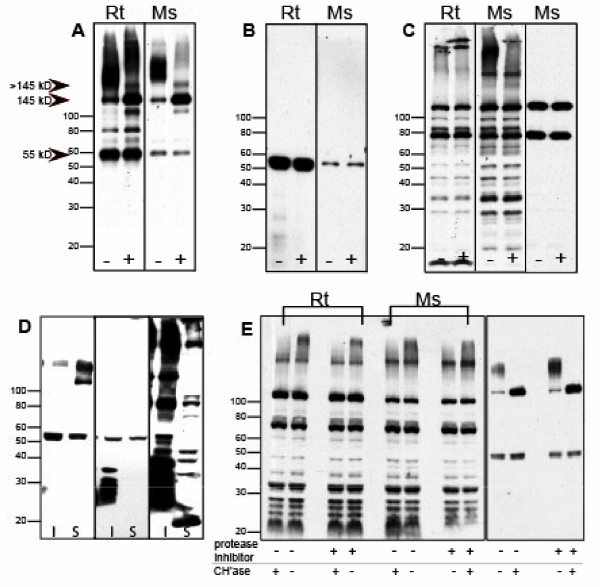
**Comparison of brevican, the ADAMTS-derived neoepitope of brevican and WFA reactivity in rat and mouse brain extracts**. Western blot of brevican, EAV(M)ESE, and *Wisteria floribunda *agglutinin (WFA) in rodent brain extracts before and after chondroitinase digestion: Rat (Rt) and mouse (Ms) extracts were probed for (A) anti-brevican, (B) anti-EAVESE (Rt) or anti-EAMESE (Ms), or (C) biotinylated WFA: Samples were treated with (+) and without (-) Chondroitinase ABC (Chase). (A+) The 145 kD core protein of brevican increased after Chase treatment, (B+) the proteolytic brevican fragment remained unchanged, and (C+) only a high molecular weight WFA-reactive band was diminished in Ms. (C) After probing with WFA, multiple, unidentified lower molecular weight bands were observed along with less abundant, high molecular weight moieties. The right panel in (C) was probed with secondary, HRP-conjugated streptavidin alone, which revealed two, major non-specific bands. (D) After differential centrifugation of rat brain tissue, brevican immunoreactivity (left panel) was predominately found in the soluble fraction (S), whereas most of the WFA reactivity (right panel) was observed in the membrane "insoluble" fraction (I) whereas anti-EAVESE immunoreactivity (middle panel) was evident in both fractions. (E) Rt and Ms samples were treated with Chase in the absence (-) and presence (+) of a protease inhibitor cocktail (left panel) and probed with biotinylated-WFA. The high molecular weight smear is eliminated after treatment with Chase, but the protease inhibitor did not change the pattern. (right panel) The same membrane was probed with anti-brevican where complete removal of CS chains led to an increase in abundance of the core protein with no change in the abundance of fragment. Protease inhibitor had no effect on Chase action.

In an effort to identify the molecular species in rodent brain detected by the N-terminal region, anti-brevican antibody, those detected by anti-EAV(M)ESE, and by biotin-WFA, cortical homogenates from rat and mouse brain that were either pre-treated with Chase or were left untreated were subjected to SDS-PAGE and probed with the reagents (Fig. 3). When soluble brain extracts were probed with the N-terminal region anti-brevican antibody, the pattern of brevican immunoreactivity included a smear of immunoreactivity found at > 145 kD, a distinct 145 kD band and a 55 kD, N-terminal fragment of brevican (Fig. 3A). Chase treatment eliminated the smear in the mouse sample with an associated increase in the 145 kD holoprotein without CS in both the mouse and rat samples (Fig 3A). Chase treatment did not alter the intensity of the 55 kD fragment, and this same N-terminal fragment was identified (alone) when the membrane was probed with anti-EAVESE (rat) or anti-EAMESE (mouse) (Fig. 3B). Interestingly, signals observed with streptavidin-HRP to identify proteins on blots that bind to biotin-WFA did not correspond to the molecular weight of any isoform of brevican. In fact, the very high molecular weight smear observed in cortical extract was the only WFA-reactive species modestly diminished after incubation with Chase (Ms, Fig. 3C). Two of the major bands seen in this blot, however, were non-specific binding signals that were present when membrane was probed with streptavidin-HRP alone (Fig. 3C, right lane, Ms). When brain homogenates were differentially centrifuged to obtain "membrane" and "soluble" fractions, the majority of brevican immunoreactivity was found in the soluble fraction (Fig. 3D, left panel, S), yet the generalized and ADAMTS derived fragments were found in both fractions (Fig. 3D, left and middle panel). However, the major signals observed after probing with biotin-WFA were found mainly in the "insoluble", membrane fraction (Fig. 3D, right panel, I). These results indicate that brevican isoforms were undetected when probed with biotinylated WFA on membranes. To verify that the effect of Chase on the high molecular weight, WFA reactive smear was due solely to degradation of polysaccharides (and not to proteolytic activity potentially contained within the Chase preparation), samples were treated with Chase in the absence and presence of protease inhibitor cocktail (Fig. 3E, left panel). In both rat and mouse samples, the pattern of WFA reactivity was identical, whether or not the samples contained protease inhibitor cocktail. These results suggest that WFA does indeed bind to a high molecular weight, CS moiety that is removed after Chase treatment, but that is clearly different from brevican. To verify the effectiveness of Chase digestion, the same membrane was probed with anti-brevican antibody which showed complete removal of CS chains and a marked increase in the abundance of the core protein in Chase-treated samples (Fig. 3E, right panel, rat only, mouse not shown) with no change in the abundance of fragment.

**Figure 4:**
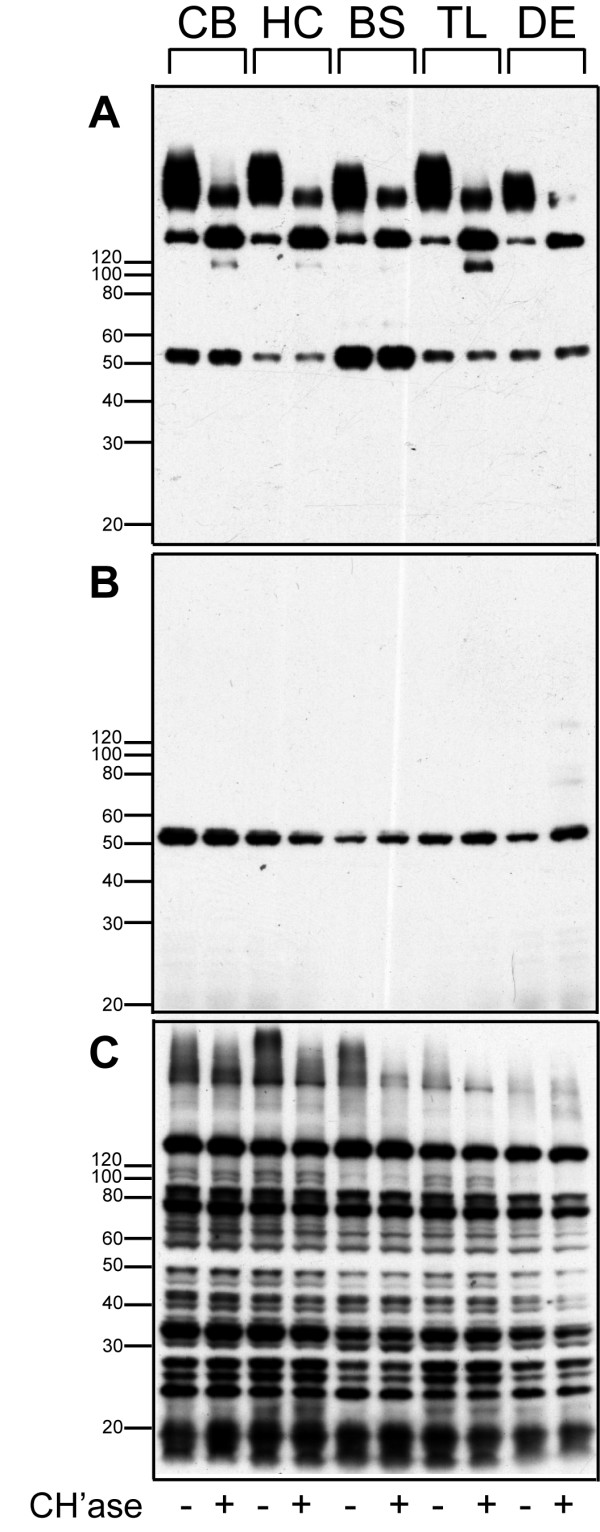
**Regional distribution of brevican, EAMESE and WFA reactivity in rat brain**. Western blot of brevican, EAMESE, and biotinylated-*Wisteria floribunda *agglutinin (WFA) in extracts from various regions of mouse brain before and after Chase digestion: Cerebellum (CB), hippocampus (HC), brain stem (BS), temporal lobe (TL) and diencephalon (DE)) extracts were probed for (A) anti-brevican, (B) anti-EAMESE and (C) biotinylated-WFA. Samples were pre-treated with (+) and without (-) Chase. (A+) The 145 kD core protein of mouse brevican increased after chondroitinase treatment, (B+) the proteolytic fragment remained unchanged, and (C+) high molecular weight, biotinylated WFA reactive smears were only slightly affected by Chase.

**Figure 5:**
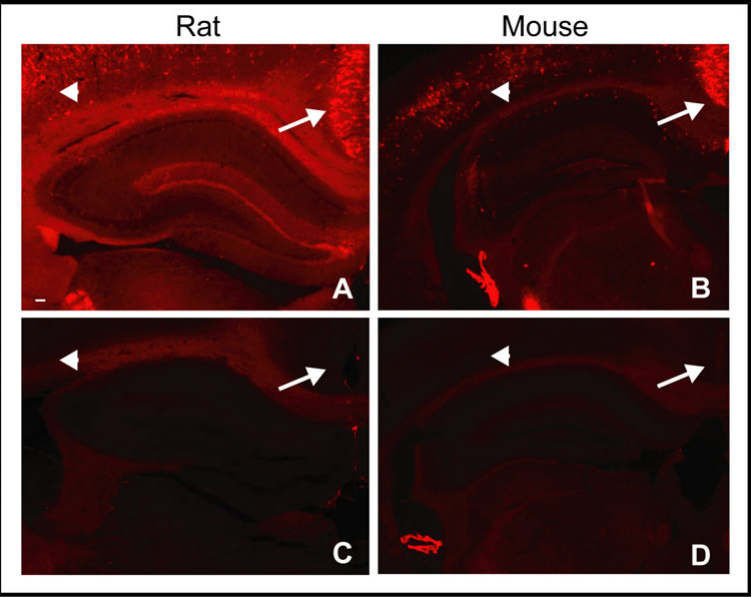
**Chondroitinase treatment of brain sections prior to binding by WFA**. Binding of *Wisteria floribunda *agglutinin (WFA) lectin after treatment with Chase. Paraformaldehyde-fixed coronal sections from (A and C) rat and (B and D) mouse brain were probed with biotinylated-WFA (A and B) before and (C and D) after Chase treatment. Arrow = retrosplenial cortex; arrow head = parietal cortex. Note the near elimination of reactivity after Chase treatment. All images were captured at 25× magnification. Scale bar represents 100 μm.

This discordance between biotin-WFA and anti-brevican reactivity was not region specific, because when cerebellum, brain stem, temporal lobe and diencephalon extracts were probed with anti-brevican, anti-EAMESE and biotin-WFA, similar results were observed (Fig. 4). Nonetheless, biotin-WFA was highly effective at identifying neurons in the CNS that were surrounded by PNNs, and minor reactivity was also found in the neuropil in both rat and mouse tissue sections (Fig. 5A, 5B). Note the abundant PNNs in retrosplenial cortex (arrows) and scattered PNNs in parietal cortex. The intense signal was nearly abolished by pre-incubating the tissue section with high concentrations of Chase (Fig. 5C, 5D). When streptavidin-Alexa 594 was incubated alone, the section was completely blank (not shown). In addition, immunoreactivity for anti-EAV(M)ESE was not influenced at all by Chase pre-treatment of a tissue section (not shown). These results suggest that whatever moiety is bound by WFA is released upon treatment with Chase, yet the N-terminal brevican fragment that does not bear CS chains is unaffected by Chase as would be predicted.

**Figure 6:**
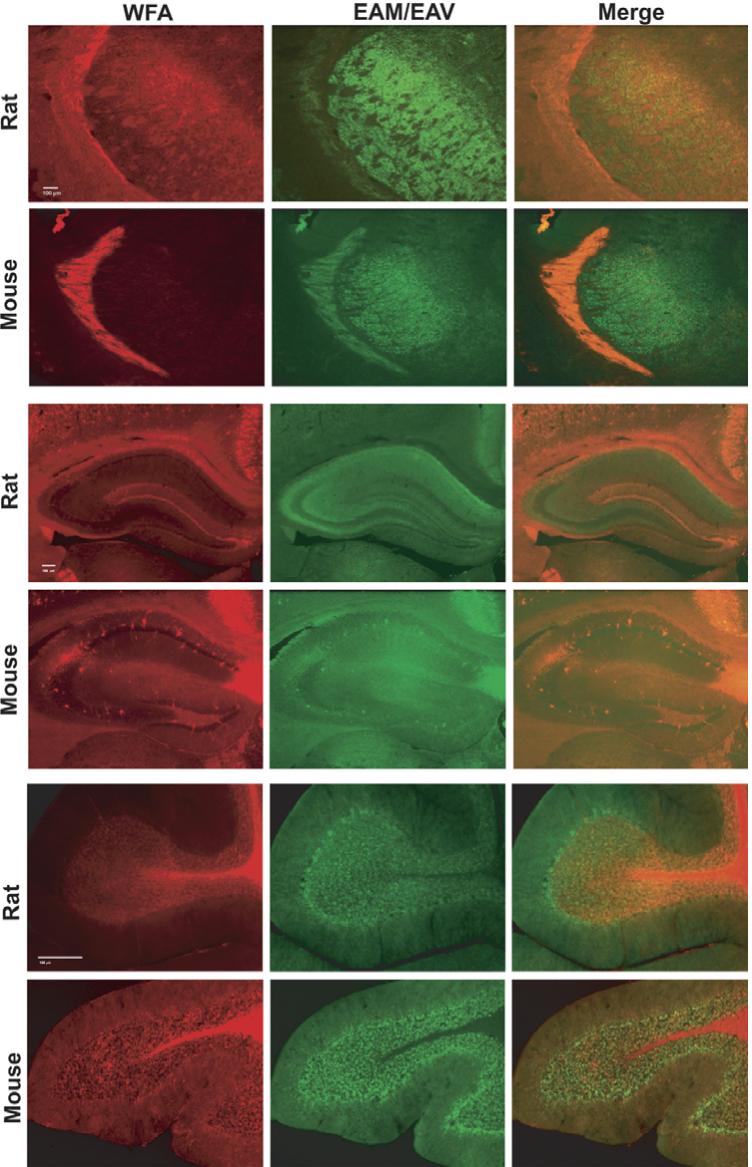
**Histochemical localization of WFA and ADAMTS-derived brevican fragment in rat and mouse brain**. Localization of *Wisteria floribunda *agglutinin (WFA) and ADAMTS-derived fragment of brevican in rat and mouse brain including lateral thalamus (A-F), hippocampus (G-L) and cerebellum (M-R): Epifluorescent micrographs of biotinylated-WFA reactivity (A, D, G, J, M, and P, red), anti-EAVESE (rat) immunoreactivity (B, H, N, green), anti-EAMESE (mouse) immunoreactivity (E, K, Q, green) and merged composites of WFA and anti-EAVESE (C, I O), and WFA and anti-EAMESE (F, L, R) in fixed brain sections. Images A-L were captured at 25× magnification and M-R were captured at 100× magnification. Scale bar represents 100 μm.

There are several regions of the brain where there is distinctive and discordant reactivity between WFA and the ADAMTS-derived N-terminal fragment of brevican. Particularly, the reticular thalamic nucleus shows prominent staining with WFA in both the rat and mouse (Fig. 6A, 6C, and 6D, 6F) whereas the barrels of the ventral posteriolateral and posteriomedial thalamic nuclei are most evident in the rat, compared to the mouse (Fig. 6A and 6C with 6D and 6F). In contrast, anti-EAV(M)ESE immunoreactivity is weak in the reticular thalamus, but highly prominent between the barrels in the posteriolateral and medial nuclei (Fig. 6B, 6C, 6E and 6F). There were also striking differences seen in the hippocampus and surrounding cortex as well. Mostly the neuropil layers of the dorsal hippocampus contain weak to absent staining with WFA, the exceptions being the CA2-CA3 transition region, the molecular layer of the lateral blade and the polymorphic layer (especially in the mouse) of the dentate gyrus and the fasciola cinereum (Fig. 6G). However, clear PNNs were found in and just adjacent to the pyramidal cell layer in the stratum oriens in Ammon's horn. These hippocampal neurons containing PNNs were more abundant in the mouse compared to the rat (Fig. 6G, 6J). In addition, the white matter regions of the corpus callosum, external capsule and alveus were all intensely stained by WFA; however, at least a portion of this staining was not eliminated after Chase treatment of the sections (not shown). Staining by anti-EAV(M)ESE showed comparatively intense immunoreactivity in the hippocampal neuropil, especially the stratum oriens, and this staining reached the pyramidal cell layer (Fig. 6H, 6K) where there was a cobblestone appearance of this layer (not shown). Scattered PNNs were noted in and around the pyramidal cell layer, similar to WFA staining, and some of these appeared to co-localize with WFA reactivity in the mouse (Fig. 6J, 6K, 6L). In the cerebellum, the lobular molecular layer was weakly stained by WFA, the granular layer contained heavily labeled neuropil and PNNs and lobule white matter (Fig. 6M, 6P). The white matter was perfectly negative for reaction product to EAV(M)ESE, however, marked immunoreactivity was observed in the granular layer, especially as PNNs surrounding the aligned Purkinje cells that make up the molecular-granular layer interface (Fig. 6N, 6O, 6Q, 6R). Interestingly, these aligned Purkinje neurons have previously been shown to be labeled with parvalbumin but not by WFA [27].

**Figure 7:**
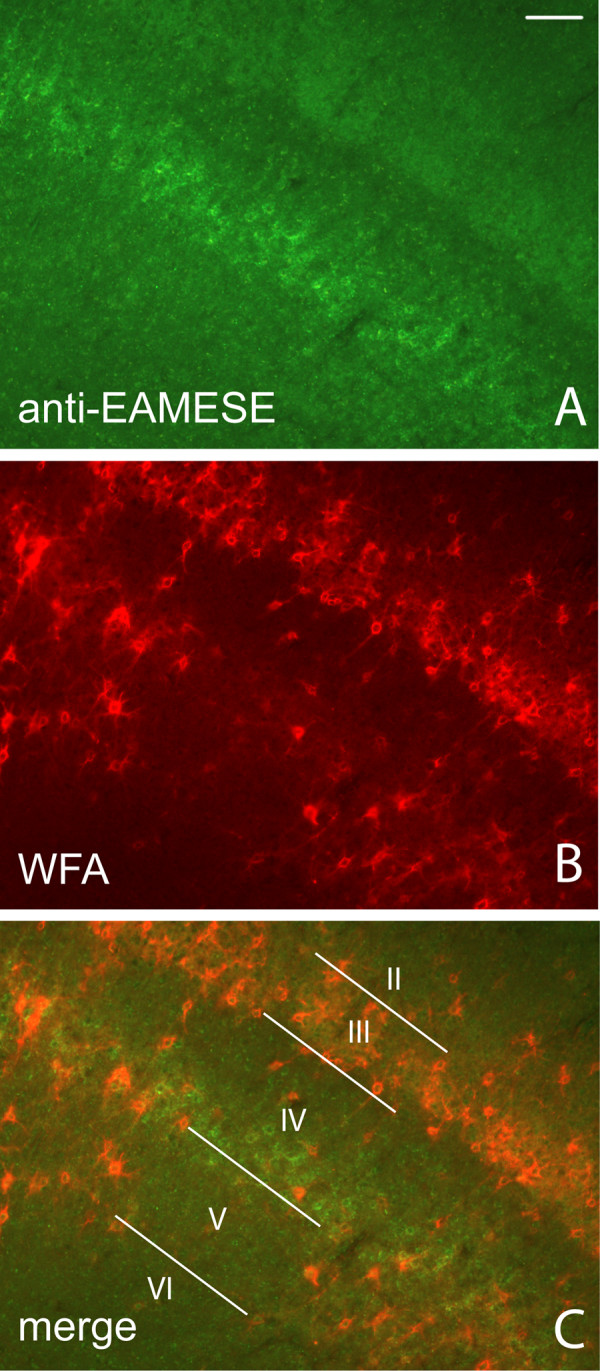
**Identification of PNNs with antibody against the ADAMTS-derived fragment of brevican**. Mouse PNNs immunoreactive for the ADAMTS-derived fragment of brevican distinguished with anti-EAMESE (green) (A) were broadly distributed in cerebral cortex, but were especially prominent deep in cortical layer IV, which differs from the distinct pattern of WFA staining of PNNs (B). The most intense region of WFA reactivity appears in cortical layer III of primary somatosensory cortex (B-C). Scattered WFA positive PNNs are also found in layer V. Images A-C were captured at 100× magnification. Scale bar represents 100 μm.

Neurons with PNNs that contain EAV(M)ESE fragment are not quite as abundant as those identified by WFA, but they appear to have a broad distribution. PNNs containing the brevican fragment were broadly distributed in cerebral cortex in layers distinct from PNNs surrounded by WFA reactivity, (Fig. 7A – 7C). In murine cerebral cortex, anti-EAMESE immunoreactivity was found mostly in deep cortical layer IV where sporadic neurons containing WFA-reactive PNNs were located. For WFA reactivity, an intensely-stained region was primary somatosensory cortex (Fig. 7B – 7C). However, most neurons with WFA reactive PNNs were found in layer III, and the superficial region of layer IV was deficient in PNNs positive for WFA or anti-EAMESE (Fig. 7F). Both reagents show a similar distribution in the rat (data not shown). Another region of cortex that is intensely labeled by both WFA and anti-EAV(M)ESE was retrosplenial cortex (at low magnification, see Fig. 6G – 6L). Anti-EAVESE immunoreactive PNNs and intense fiber staining were found in the horizontal limb of the diagonal band, the medial septum and in piriform cortex (not shown).

**Figure 8:**
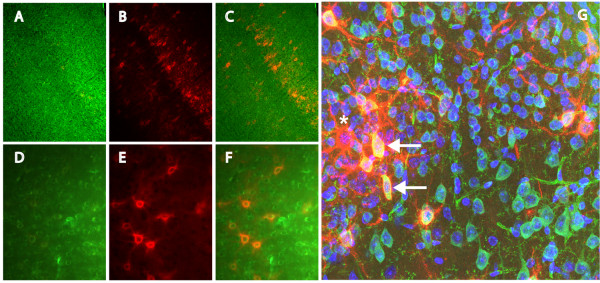
**Localization of WFA and brevican reactivity in PNNs**. Brevican immunoreactivity was found in (A and C) neuropil and PNNs of cortical layers II, III, deep layer IV and V. WFA immunoreactivity is predominant in (B and C) PNNs of cortical layer III. A higher magnification of cortex reveals PNNs that are positive for (D) brevican and (E) WFA reactivity. A confocal micrograph (G) of retrosplenial cortex stained with anti-brevican (green), biotinylated-WFA (red) and DAPI (blue) demonstrates a small subpopulation reactive for both anti-brevican and WFA (arrows). While the majority of PNNs showed only brevican immunoreactivity, some were identified as only WFA reactive (denoted by asterisk). Images A-C were captured at 100× magnification, D-F were captured at 200× magnification and confocal image G was captured at 630×. Scale bar represents 100 μm.

Immunoreactivity for brevican holoprotein was distributed throughout the CNS neuropil and in PNNs as has been identified by others [7]. Neurons with PNNs that contain brevican immunoreactivity are clearly more abundant than those identified by WFA. Brevican immunoreactivity was broadly distributed in cerebral cortex in layers distinct from PNNs surrounded by WFA reactivity, (Fig. 8A – 8C), although there was a higher percentage of brevican immunoreactive cells that co-localize with WFA compared to that of EAV(M)ESE immunoreactivity. In murine cerebral cortex, anti-brevican immunoreactivity was found in neuropil and PNNs of cortical layers II, III and deep layer IV and V, whereas the most abundant distribution of neurons containing WFA-reactive PNNs were found in layer III. Both reagents showed a similar distribution in the rat (data not shown). A higher magnification of cortex reveals PNNs that are positive for brevican and WFA reactivity (Fig. 8D – 8F). A confocal micrograph of retrosplenial cortex stained with anti-brevican, biotin-WFA and DAPI, demonstrated that there are clearly PNNs that co-localize and are reactive for both reagents (Fig. 8G – denoted by arrows). While many PNNs were immunoreactive for brevican, other PNNs were reactive toward WFA alone, (Fig. 8G – denoted by asterisk). PNNs reactive with WFA and not brevican, expressed other CS-containing PGs or N-acetylgalactosamine containing molecules. A complete localization of anti-brevican, anti-EAV(M)ESE, and biotinylated WFA PNNs and their reactivity in the neuropil of the CNS, with semi-quantitation for both reagents may be found in additional files [Additional file 1] (rat) [Additional file 2] (mouse).

## Discussion

Recent evidence indicates that ECM molecules are important regulators of structural neural plasticity, and regional expression in the CNS may be indicative of their role. Localization of brevican by immunohistochemistry [7,28] and *in situ *hybridization [29,30] within the CNS revealed high expression in cerebellar and cerebral cortex, hippocampus and thalamic nuclei and brain stem. It is localized perisynaptically, it inhibits neurite outgrowth [31] and it is thought to stabilize neural networks in the adult [13]. In contrast, conditions that augment the proteolytic cleavage of brevican, especially ADAMTS-induced cleavage, are associated with enhanced neural plasticity [16,24,32]. Here, an antibody which selectively recognizes the neoepitope sequence of brevican that is uncovered upon proteolysis by an ADAMTS was used to map the distribution of this fragment in untreated rodent CNS. This pattern was compared to the distribution of PNNs and neuropil stained by the classical reagent WFA. By both Western blot and immunochemistry, the ADAMTS-derived fragment of brevican appeared to be stable, abundant and widely-distributed. There was a discordance between the regional and local expression of classical PNNs identified by WFA and regions where the proteolytic fragment of brevican was most highly expressed. In addition, there appeared to be an association between regions with significant deposition of the brevican fragment and areas known to be involved in neural plasiticity (eg. hippocampus), supporting the involvement of ADAMTSs in neural plasticity mechanisms [16,21,24]. WFA staining in these regions was notably low.

A significant proportion of total brevican immunoreactivity in brain extracts was found as a fragment formed by proteolytic cleavage of the intact holoprotein. In fact, all antibodies raised against the brevican holoprotein, intended only to detect the holoprotein, inherently recognize either the N- or C-terminal proteolytically-cleaved fragment. Proteolysis of lecticans may be an important mechanism by which the nervous system overcomes inhibition exerted by PGs during periods of neural plasticity. After systemic injection of the excitotoxin, kainic acid [16] or after discrete, unilateral lesion of the entorhinal cortex [24], there was an increase in the abundance of an ADAMTS-derived brevican fragment in the dentate gyrus, the target of projections from entorhinal cortex and a region where sprouting occurs in response to the lesion. Thus, proteolytic cleavage of PGs may be a key mechanism involved in sprouting and reorganization of the dentate gyrus, whereas intact PGs may promote neural stabilization.

There is debate in the literature about which molecule(s) in the CNS are labeled by WFA, and here we investigated whether WFA binds to the CS-bearing lectican, brevican. WFA is a lectin that binds to N-acetylgalactosamine-linked α or β to the 3 or 6 position of galactose [33]. WFA is often used as marker for PNNs that contain CS chains, yet only indirect evidence suggests that WFA binds to CS polymers. Our data show that WFA clearly did not recognize CS-containing brevican on Western blot of soluble brain extracts, although there was at least one high molecular weight WFA reactive band that was diminished upon treatment with Chase. No corresponding bands were observed at the molecular weights representing any isoform of brevican. In addition, the preponderance of brevican was found in the soluble fraction of brain extract, whereas WFA reactivity was mainly located in the particulate, insoluble fraction after differential centrifugation of brain extract. These results reveal that WFA binds molecules that differ from the lectican, brevican, although the possibility that WFA has a differential affinity for selected sulfated forms of CS cannot be excluded. Murakami et al has gathered convincing data that indicates that WFA binds to cell surface glycoproteins. This group showed that terminal N-acetylgalactosamine residues, which are present on neuronal cell surface glycoproteins, are responsible for the PNN reactivity seen with WFA lectin binding [9,34]. Based on a series of studies using degradative enzymes to define the presence or absence of polysaccharides bound to ECM proteins, their model suggests that perineuronal proteoglycans, such as brevican, bind to these cell surface glycoproteins. Chase treatment removes the terminal N-acetylgalactosamine from these glycoproteins, thereby releasing the lectican from its binding partner on the cell surface. This may, in part, account for the discordance in reactivity seen here between brevican and WFA.

No direct evidence exists, to our knowledge, of WFA binding to any specific CS-containing PG, including the lecticans. However, the reactivity observed with WFA and antibodies generated against CS chains are similarly distributed in rat brain [8] (our unpublished observations), such as in PNNs, and binding of WFA to fixed brain sections is diminished or lost after Chase treatment [35,11]. Nonetheless, there may be a host of proteins present in ECM complexes and it is possible, in fact likely, that WFA binds to a molecule that may be indirectly bound to CS. In any case, no biochemical evidence is available to show that WFA binds to large polymeric chains of repeating disaccharides that contain N-acetylgalactosamine.

The differential distribution between WFA and the fragment of brevican may relate to their functional environment, i.e. greater abundance of intact CS-bearing PGs chains in regions stained with WFA (more stable environment) whereas increased in proteolytic cleavage stained the neoepitope fragment in regions that are capable of undergoing neural plasticity. These findings are intriguing since we observe intense immunoreactivity for the ADAMTS-derived brevican fragment in areas thought to be highly plastic such as the hippocampus. Therefore, the relative abundance of cleaved proteoglycans, such as brevican, in a particular region suggest a functional change in surrounding ECM complexes which may contribute to overall neural plasticity. However, the data presented here only begin to uncover the intricate molecular environment around individual neurons that modulate their function and structure.

## Conclusion

A marked discordance was observed in the regional localization between WFA and brevican or the proteolytically-derived N-terminal fragment of brevican. Functionally, this difference may correspond to regions with varied prevalence for neural stability/plasticity.

## Methods

### Animals

All animal procedures described in this manuscript were approved by the Institutional Animal Care and Use Committee (IACUC) at the University of South Florida. Adult male C57BL/6 mice (23 g – 27 g; Harlan, Indianapolis, IN) and adult male Sprauge-Dawley rats (250 g – 300 g; Harlan, Indianapolis, IN) were housed under a 12 hour light cycle with regulated temperature and humidity. Mice were housed 3 to 4 per cage and rats were housed individually with both having free access to food and water. Brain tissue was collected from animals between 3 and 4 months of age: For biochemical analysis n = 4 and immunohistochemistry n = 6.

### Western Blotting

For collection of tissue immunoblotted with antibodies and biotin-WFA, animals were euthanatized by exposure to excess CO_2 _until death and immediately decapitated. Various brain regions were rapidly dissected and extracted with a teflon-glass homogenizer in 5 volumes of Triton-X-100-containing buffer (20 mM Tris-HCl at pH 7.4, 10 mM EDTA, 1% Triton-X-100, and 1:100 protease inhibitor cocktail [Calbiochem type III, LaJolla, CA]) for 2 minutes. The homogenate was centrifuged in a microcentrifuge at 6800 × g for 5 minutes, and the supernatant collected and stored at -80°C.

In some experiments, brain tissue extract was treated with Chase prior to Western blot to determine whether WFA recognized CS-containing proteins. Thus, 25 μl of sodium acetate buffer (50 mM sodium acetate, 1 M Tris, 10 mM EDTA) containing 10 mU of chondroitinase ABC (Sigma-Aldrich, St. Louis, MO) was added to 25 μl of brain tissue extract and incubated for 1.5 h at 37°C. To determine whether there was protease contamination in the Chase preparation, samples underwent Chase digestion in the presence of a protease inhibitor cocktail. All samples were reduced (mercaptoethanol-containing, SDS-PAGE sample buffer), denatured for 4 minutes at 95°C, and subjected to SDS-PAGE.

Tissue extracts were loaded (equal amounts of protein) onto pre-cast, 1.5 mm, 4–20% gradient SDS-PAGE gels (Novex Tris-glycine, Invitrogen, Carlsbad, CA). Separated proteins were electrophoretically transferred to a polyvinylidine difluoride membrane (PVDF, Immobilon, Millipore, Billerica, MA). For brevican and EAV(M)ESE immunoblotting, the membranes were washed with Buffer B (10 mM phosphate buffered saline, pH 7.4 containing 0.05% Tween 20) for 5 minutes, blocked for 1 h in 5% non-fat dry milk diluted in Buffer B and probed for 2 hours using primary antibodies against mouse anti-brevican (1:1000, BD Transduction Labs, San Jose, CA), rabbit anti-EAMESE (1:1000) [24] (the neoepitope sequence for mouse brevican fragment), or rabbit anti-EAVESE (1:500) [23,36] (the neoepitope sequence for rat brevican fragment). For WFA blotting, the membranes were washed with Buffer B for 5 minutes, blocked in 1% bovine serum albumin diluted in Buffer B for 1 hour and probed for 2 hours using biotinylated *Wisteria floribunda *lectin (1:10,000 in 1% BSA, Vector Laboratories, Burlingame, CA) as the primary binding reagent. Primary antibodies and biotinylated *Wisteria floribunda *lectin were detected with corresponding secondary antibodies including anti-mouse, anti-rabbit and streptavidin conjugated to horse radish peroxidase (Chemicon, Temecula, CA), respectively. Antigens were visualized using a chemiluminescence developing system (SuperSignal, Pierce, Rockford, IL), and equal protein loading was verified by examining the Commassie blue stained membrane. It should be noted that ADAMTS-derived fragment antibodies were raised against the species-specific neoepitopes for rat and mouse, since they show limited cross-reactivity with one another, ie. anti-EAVESE (rat sequence) does not effectively recognize the C-terminus of the N-terminal, ADAMTS-cleaved fragment EAMESE (murine sequence) of mouse brevican.

### Isolation of Membrane Fractions

Whole rat brain was collected as descibed above and homogenized for 1 minute with a Glas-Col (Terre Haute, IN) motorized (low speed, 333 rpm), teflon-glass homogenizer in 10 volumes of 50 mM Tris-HCl, pH 7.4, 1 mM EDTA, containing 1:100 protease inhibitor cocktail (Calbiochem type III, LaJolla, CA). The homogenate was centrifuged at 500 × g for 5 minutes, the supernatant collected and centrifuged at 40,000 × g to obtain soluble and insoluble fractions. The supernatant, "soluble" fraction, was removed immediately, aliquoted and stored at -80°C. The insoluble "membrane" fraction was resuspended with buffer, centrifuged again (40,000 × g for 30 min) and reconstituted in detergent-containing RIPA buffer (50 mM Tris base, 150 mM NaCl, 1 mM EDTA, 1 mM EGTA, 1% Triton-X-100, 1% sodium deoxycholate, 1% SDS, pH = 7.4), aliquoted and stored at -80°C.

### Immunohistochemistry

Rats and mice were euthanatized with excess Nembutal, and the brains fixed via cardiac perfusion as described [36]. Brains were cleared using phosphate buffered saline (PBS; pH 7.4), fixed with fresh 4% paraformaldehyde in 0.1 M phosphate buffer (PB; pH 7.4), collected, post-fixed overnight in 4% paraformaldehyde and cryoprotected with 15% and 30% sucrose (in PBS) for 24 h each. The individual brains were mounted on a cryostat chuck at -20°C and sectioned at 30 μm. Sections were stored freely floating in antifreeze solution (50 mM sodium phosphate, pH 7.4, 30% ethylene glycol and 30% glycerol) at -20°C.

For Chase treated tissue, matched sections were selected, washed three times with PBS and incubated in 500 mU of Chase in 0.5 ml of sodium acetate buffer for 1.5 hours at 37°C. Selected sections to be used for immunohistochemistry were washed in PBS for 15 minutes, blocked and permeabilized in 10% normal goat serum, 3% 1 M lysine and 3% Triton-X-100 for 1 h and incubated overnight in primary antibodies anti-EAMESE (1:1000) (Mayer et al., 2005), EAVESE (1:500) [16,36], brevican (1:1000, N-terminal (G1); Transduction Labs, San Jose, CA), RB18 (1:500, a generous gift from Yu Yamaguchi, Burnham Institute, La Jolla, CA, an antibody that recognizes an epitope in the G3 domain of rat brevican) and *Wisteria floribunda *lectin (1:1000) at 4°C. Doubly probed sections were washed and incubated in anti-rabbit IgG conjugated to Alexa-Fluor 488 (Molecular Probes, Eugene, OR) and streptavidin conjugated to Alexa-Fluor 594 (Molecular Probes, Eugene, OR) for 1 hr at room temperature. The sections were washed for 15 minutes, wet mounted on glass slides, and coverslipped with VectaShield mounting medium (Vector Labs, Burlingame, CA).

### Cleavage of PGs with human recombinant ADAMTS4

PGs present in whole rat brain extracts were bound to and eluted from a DEAE Sepharose Fast Flow cation exchange matrix (Pharmacia, Pfizer, New York, NY) as described [37] with modifications. All procedures were carried out at 4°C unless otherwise stated. Briefly, rat brain tissue (1 g) was placed in 10 ml, ice cold, 4 mM HEPES pH 8.0, 0.15 mM NaCl, 0.1% Triton-X-100 containing 2 mM 1,10 phenanthroline (Sigma, St. Louis, MO) and protease inhibitor cocktail (set III, Calbiochem, San Diego, CA). The tissue was disrupted in a Teflon-glass homogenizer and the extract centrifuged at 30,000 × g for 30 min. The supernatant was removed, diluted 1:1 with 50 mM Tris-HCl, 0.15 M NaCl, 0.1% Triton-X-100, and applied to a DEAE column pre-equilibrated with the same buffer at a flow rate of less than 0.5 ml per minute. The flow through was collected, passed over the column again, and bound proteins were eluted with 5 column volumes of consecutive buffers containing 50 mM Tris-HCl pH 8.2, 0.15 M NaCl, 0.1% Triton-X-100, then 50 mM Tris-HCl pH 8.2, 0.25 M NaCl, 6 M urea, 0.1% Triton-X-100, and fractions containing PGs were eluted with 50 mM Tris-HCl pH 8.2, 1.0 M NaCl. PG (protein)-containing fractions were dialyzed against water for 24 h in SpectraPor 6000–8000 MWCO (Millipore, Billerica, MA) membrane at 4°C, the samples concentrated on an Eppendorf "speed-vac" and aliquoted. Total protein was measured in the samples (1.3 μg/μl). DEAE-purified PG samples were incubated with 25 nM human recombinant ADAMTS4, (a gift of Carl Flannery, Wyeth Pharmaceuticals, Collegeville, PA) diluted in 10 mM Tris-HCl, 0.15 M NaCl, and 10 mM CaCl_2 _for two hours at 37°C. After the incubation period, beta-mercaptoethanol-containing, SDS-PAGE sample buffer was added to the samples, the samples were heated at 95°C for 4 minutes, subjected to SDS-PAGE, and electrically transferred to Immobilon PVDF membrane (Millipore, Bedford, MA). Membranes were probed with mouse anti-brevican (BD Biosciences, San Jose, CA) at 1:1000 primary antibody detected with anti-mouse conjugated to horse-radish peroxidase (Chemicon, Temecula, CA), and signal detected using SuperSignal chemiluminescence substrate (Pierce, Rockford, IL). The membrane was probed a second time with anti-EAVESE (1:100) [23], as described under the Western blot section above.

### Microscopy and image acquisition

Single and multi-labeled, epifluorescent tissue sections were viewed using a Zeiss Axioskop microscope, interfaced with an Axiocam and images acquired with Openlab software. Confocal images (Fig. 8) were attained using a Leica SP-2 confocal microscope and Leica LCS software. Controls for each immunomarker included secondary antibody in the absence of a primary antibody, in which the staning in control sections was minimal to absent. Exposure times and aperture opening were constant for each magnification and antibody used. Some images were minimally and equally modified (contrast and brightness) using Abobe photoshop.

## List of abbrevications

PG – proteoglycan, ECM – extracellular matrix, CNS – central nervous system, CS – chondroitin sulfate, ADAMTS – a disintegrin and metalloproteinase with thrombospondin motifs, WFA – Wisteria floribunda, PNN – perineuronal net, Chase – Chondroitinase ABC, IACUC – Institutional animal care and use committee, PVDF – polyvinylidine difluoride, MWCo – molecular weight cutoff.

## Authors' contributions

JMA was responsible for collecting the majority of data contained within the manuscript, including animal handling, tissue processing, data analysis and contributed to writing this manuscript. AKE was highly involved in semi-quantitation of the signal on brain sections for Tables 1 and 2, and performed much of the immunohistochemistry. MGH was particularly important for assisting in the experiment of Figure 2 and contributed significant intellectual input. PEG contributed to the original idea for the expeirment, discussed the data with the group on a regular basis, and was responsible for the final written version of the document. All authors read and approved the final version of the manuscript.

## Supplementary Material

Additional file 1 Relative reactivity of Wisteria floribunda agglutinin (WFA), brevican, and the neoepitope of the ADAMTS-derived fragment of brevican, anti-EAVESE, in the adult rat. This data was derived from sections at the striatal and hippocampal rostral-caudal level of male adult rat brain. Signals generated by the three reagents (WFA, anti-EAVESE) and anti-brevican) were evaluated and semi-quantitated by a single observer (AKE). Click here for file

Additional file 2 Relative reactivity of Wisteria floribunda agglutinin (WFA), brevican, and the neoepitope of the ADAMTS-derived fragment of brevican, anti-EAVESE, in the adult mouse. This data was derived from sections at the striatal and hippocampal rostral-caudal level of male adult mouse brain. Signals generated by the three reagents (WFA, anti-EAVESE) and anti-brevican) were evaluated and semi-quantitated by a single observer (AKE).al file 2 Click here for file
